# Infant Feeding Practices and Nut Allergy over Time in Australian School Entrant Children

**DOI:** 10.1155/2012/675724

**Published:** 2012-07-03

**Authors:** Jessica Paton, Marjan Kljakovic, Karen Ciszek, Pauline Ding

**Affiliations:** Academic Unit of General Practice, Australian National University Medical School, P.O. Box 11, Woden, Canberra, ACT 2606, Australia

## Abstract

*Aim*. To measure the association between infant feeding practices and parent-reported nut allergy in school entrant children. *Method*. The Kindergarten Health Check Questionnaire was delivered to all 110 Australian Capital Territory (ACT) primary schools between 2006 and 2009. Retrospective analyses were undertaken of the data collected from the kindergarten population. *Results*. Of 15142 children a strong allergic reaction to peanuts and other nuts was reported in 487 (3.2%) and 307 (3.9%), children, respectively. There was a positive association between parent reported nut allergy and breast feeding (OR = 1.53; 1.11–2.11) and having a regular general practitioner (GP) (OR = 1.42; 1.05–1.92). A protective effect was found in children who were fed foods other than breast milk in the first six months (OR = 0.71; 0.60–0.84). *Conclusion*. Children were at an increased risk of developing a parent-reported nut allergy if they were breast fed in the first six months of life.

## 1. Introduction

Peanut sensitisation and allergy in children is increasing both in incidence and prevalence in various parts of the world [1–3] although no studies to date have demonstrated this trend in Australian children. 

The evidence of the role of infant feeding practices in protection against, or causation of, peanut allergy is inconclusive. Despite breast feeding being recommended as the sole source of nutrition for the first 6 months of life [4], an increasing number of studies have implicated breast feeding as a cause of the increasing trend in nut allergy [5–7]. 

The timing of introducing complementary foods, including foods and/or fluids other than breast milk to infants, has changed over the last 50 years. In the 1960s, most infants had been exposed to complementary foods by 4 months of age. By the late 1990s, expert guidelines recommended delayed introduction of complimentary foods such as solids until after 6 months of age [8, 9]. Delayed introduction of complimentary foods has been challenged by recent population studies which suggest that the current practice of delaying complementary foods until after 6 months of age may increase rather than decrease the risk of allergy [10–15].

Understanding how infant feeding practices might influence the risk of children developing nut allergy is of particular importance given that peanut allergy accounts for two-thirds of all fatal-food induced anaphylaxis [16]. 

This study measures the association between infant feeding practices and peanut (and other nut) allergies in school entrant children in the Australian Capital Territory (ACT) between 2006 and 2009. 

## 2. Method 

All new entrants to primary schools with parent-reported nut allergy were selected from those who took part in the ACT Kindergarten Health Check between 2006 and 2009. The Health Check Questionnaire (HCQ) has been described elsewhere [17]. Data are collected on the child's demographics and parents are asked to report on a variety of health issues in their child. Of the 17401 HCQ sent between 2006 and 2009, 15258 (88%) HCQ were completed and parents of 15142 children consented to the data being used for research purposes.

A positive response to the question “Has your child ever had a strong allergic reaction to peanuts/peanut products, and/or other nuts/nut products?” in the HCQ was termed as parent reported nut allergy.

Parents reporting their child was breast fed were asked, “What age was your child when breast feeding ceased?” Parents were also asked: “Was your child fed any fluids or food, other than breast milk for the first 6 months?” and (*parents were asked to tick all that apply*) “Fluids and liquids offered in the first 6 months were: (i) water, (ii) fruit juices, (iii) baby cereal, (iv) vegetables, (v) formula milk, (vi) other...”

The prevalence of peanut and other nut allergy reported in the HCQ was estimated with binomial-based 95% confidence intervals (CIs). Demographic characteristics and infant feeding practices of children with and without reported nut allergies were compared using chi-square tests or regression models with adjustment for age and sex. Clean, nonidentifiable data were analysed using Statistical Package for Social Sciences (SPSS) (version 17.0). The ACT Department of Health Research Ethics Committee approved this study.

## 3. Results

The HCQ was delivered to all 110 primary schools in the ACT. Parents of 15142 children completed the HCQ and consented to the data being used for research purposes. Parents reported that 487 children had allergy to peanuts and 592 to peanuts and/or other nuts. The overall prevalence of reported nut allergy was estimated as 3.9% (95% CI: 3.6–4.2%) and the prevalence of reported peanut allergy was 3.2% (95% CI: 2.9–3.5%). 

13422 (88.6%) children were breast fed (1.4% unknown). The mean duration of breast feeding was 9.9 months (95% CI: 9.82–10.08).

9896 (65.4%) children were fed food and/or fluids other than breast milk in the first six months (2.2% unknown). Of these children, 46.3% were given baby cereal, 46.4% formula milk, 33.5% vegetables, 13.5% fruit juices, and 13.7% other foods. Children who were exclusively breast fed were more likely to have a nut allergy (OR = 1.43; 1.21–1.69; *P* = 0.000), whilst nut allergy was less likely to occur in children only fed food and/or fluid other than breast milk (OR = 0.63; 0.45–0.89; *P* = 0.009) and those who were both breast fed and given other food and/or fluid (OR = 0.83; 0.70–0.98; *P* = 0.025).

Comparative data on infant feeding practices and nut allergy are presented in Table 1.

Children with nut allergy were more likely to have been breast fed (OR = 1.53; 1.05–1.92; *P* = 0.010) and have a regular GP (OR = 1.42; 1.05–1.92; *P* = 0.023) than those with no allergy. Protection against nut allergy was found in children who were fed food and/or fluids other than breast milk before six months (OR = 0.71; 0.60–0.84; *P* = 0.000) (Table 2).

**Figure 1:**
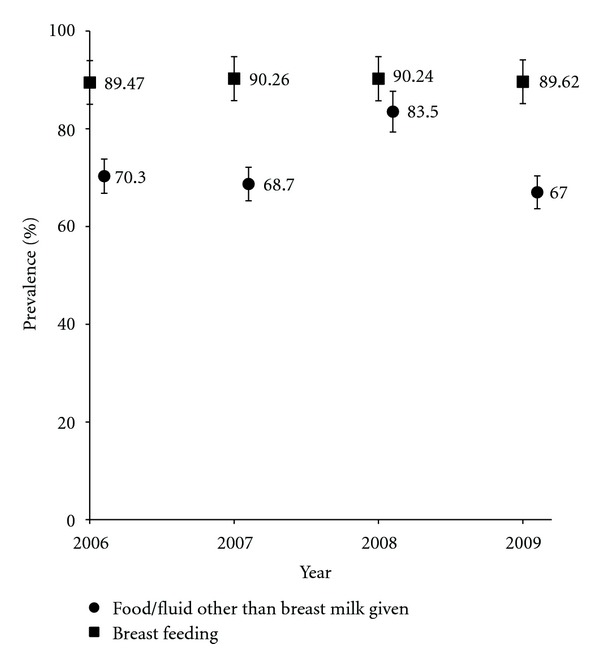
Infant feeding practices between 2006 and 2009.

**Figure 2:**
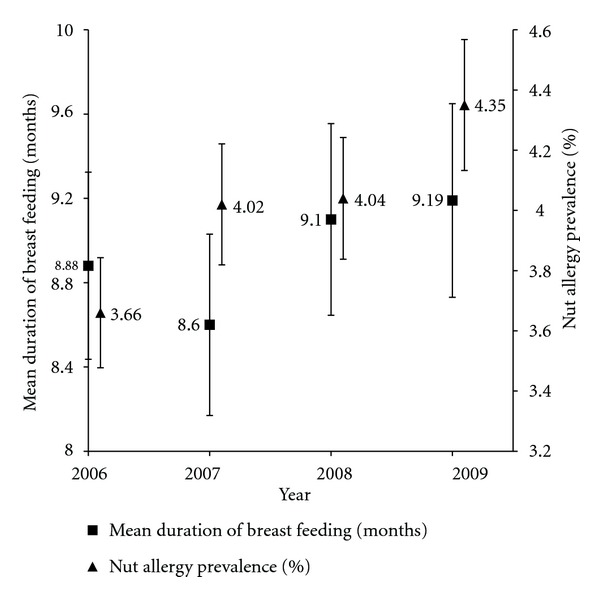
Nut allergy prevalence and mean duration of breast feeding between 2006 and 2009.

Infant feeding practices remained relatively unchanged between 2006 and 2009. Figure 1 compares the proportion of children breast fed with the proportion of children fed food and/or fluids other than breast milk in the first six months. Figure 2 compares the prevalence of parent-reported nut allergy and the mean duration of breast feeding between 2006 and 2009.

## 4. Discussion

Our study suggests nut allergy prevalence in school entrant children is increasing in the ACT. The finding is consistent with current evidence depicting Australia as part of a global trend of increasing nut allergy [1–3]. The prevalence of nut allergy for five-year-old children in the ACT is 3.9%, with peanut allergy accounting for 3.2%, which is almost twice the prevalence of British school entrant children (1.8%) [18]. 

Australian guidelines do not recommend avoiding foods in pregnancy or lactation for preventing allergic disease in infants [19]. These recommendations are made on the best available evidence at the time of writing, however, emerging evidence has proven that maternal diet during lactation is a route of allergen exposure which may result in sensitisation [20–23]. In several studies, maternal ingestion of peanut during pregnancy or lactation was shown to increase the risk of peanut allergy [21, 22]. Furthermore, in a case study of an allergic reaction to peanut in an exclusively breast fed two-week-old boy, it was concluded that the clinical symptoms of allergy could only be explained by occult ingestion through breast milk rather than environmental exposure [5]. In a study of 122 children with nut allergy, it was noted that 83% of these children were breast fed and that over 90% of the mothers admitted to ingesting peanut during lactation [24].

Our study found that almost 90% of children were reported to have been breast fed as infants and that over 4% of these children were reported to have a nut allergy. Of the remaining 10% of children who were reported not breast fed, parent-reported nut allergy prevalence was 2.72%—the likelihood of developing a reported nut allergy was 1.5 times higher in breast fed children than in nonbreast fed children. These findings are replicated in earlier studies which also concluded that nut allergy was more likely in children who had been breast fed [25]. 

These results contribute to the argument that breast feeding by itself does not appear to be protective against nut allergy in children, and that it may in fact be causative of allergy [5–7]. 

We observed an increasing trend in prevalence of parental-reported nut allergy between 2006 and 2009 confirming the findings of previous studies [1, 2]. This may be a reflection of increased consumption of peanut and peanut products by pregnant and nursing mothers [26]. It has also been argued that peanut sensitisation may occur via other subtle environmental routes, such as the application of peanut oil containing lotions to the child or the breast feeding mother [27], however, this argument is controversial [28].

Other infant feeding habits were also analysed in this study. We found that children fed only foods other than breast milk before six months were least likely to develop a parent reported nut allergy (OR = 0.63; *P* = 0.009) compared with children who were exclusively breast fed (OR = 1.43; *P* = 0.000). Children who were both breast fed and given other foods and/or fluids before six months were also protected (OR = 0.83; *P* = 0.025). The minimisation of the occult exposure of allergen that may occur with breast milk and the unknown maternal ingestion of peanut products may explain our result [5, 20]. Our findings are consistent with several studies which concluded that early introduction (prior to 4 months age) of complementary food was associated with a reduced risk of peanut sensitisation [29] and eczema in children with allergic parents [30] and that a more diverse diet of solids before 4 months was associated with a lower risk of sensitization at 6 years [15].

Furthermore, prolonged breast feeding has been shown to increase the odds of developing peanut allergy by almost 3 times that of children who were weaned at or before 6 months [27]. Our study results concur, finding a parallel between longer breast feeding time and an upward trend in parental reported nut allergy. 

Our study results also confirmed our previous finding of a positive association between parental reported nut allergy and the child having a regular GP [25]. We speculate that children with an allergy are more likely to see a regular GP for diagnosis, follow-up, and prescription for adrenalin if the allergy is severe.

Limitations in this study include inconsistency in interpreting the words “strong reaction” in the HCQ. Parents may have interpreted the words “strong reaction” in the screening question to mean either a reaction to a diagnostic test or a clinical reaction. Also, the study design does not allow causality to be inferred. Finally, data were collected four or five years after infant feeding habits had ceased—accuracy of recall impacts upon results. A strength of this study is that the data describe the association between breast feeding and parent reported nut allergy in a sample population that is highly representative of the ACT community of school entrant children. The large sample size, number of years of data collection and high response rate has reduced the chance of random error. However, there is the possibility of over-representation of a Caucasian population in the ACT and hence extrapolation of these findings onto ethnic populations is not appropriate. 

## 5. Conclusion

Children who were breast fed in the first six months of life were at an increased risk of developing a parent-reported nut allergy. A protective effect against parent-reported nut allergy was found in children who were given food/fluid other than breast milk before six months either exclusively or in combination with breast milk. There was a positive association between parent-reported nut allergy and having a regular GP. With the rate of parent-reported nut allergy in a highly representative sample population being 1 in 25 and peanut allergy being 1 in 30, there is a need for further research in this field. The scarce and often contradicting evidence regarding nut sensitisation and infant feeding practices demands particular emphasis. 

## Figures and Tables

**Table 1:** Odds ratios for reported nut allergy and infant feeding practices of kindergarten children in the Health Check Questionnaire 2006–2009.

Infant feeding practice in the first 6 months	Reported nut allergy^∗^			Odds ratio (95% CI)^#^	*P* value
	Yes		No
Breast feeding only (*n* = 4647)	232	(5.0%)	4415	1.43 (1.21–1.69)	0.000
Other food/fluid only (*n* = 1312)	35	(2.7%)	1277	0.63 (0.45–0.89)	0.009
Breast feeding + other food/fluid (*n* = 8368)	311	(3.7%)	8057	0.83 (0.70–0.98)	0.025
Unknown (*n* = 138)	4	(2.9%)	134		

^∗^Parental response to the question: “Has your child ever had a strong allergic reaction to peanuts/peanut products, and/or other nuts/nut products?”

^#^Adjusted for age and sex where appropriate. Children with invalid data on the nut allergy and feeding practice questions were excluded from comparative analyses.

**Table 2:** Odds ratios for reported nut allergy and characteristics of kindergarten children in the Health Check Questionnaire 2006–2009.

Characteristic of child		Reported nut allergy^∗^		Odds ratio (95% CI)^#^	*P* value
		No	Yes		
Sex of child	Male	7184	322	1.15 (0.97–1.35)	0.105
Age	5 years	12257	511	1.11 (0.87–1.42)	0.385
Aboriginal or Torres Strait Islander	Yes	238	11	1.11 (0.60–2.05)	0.737
	Unknown	417	19		
Child has a usual general practitioner	Yes	12503	543	1.42 (1.05–1.92)	0.023
	Unknown	92	2		
Child was breast fed	Yes	12599	549	1.53 (1.11–2.11)	0.010
	Unknown	101	2		
Duration of breast feeding (mean 9.9 months)	—	—	—	1.02 (1.01–1.03)	0.000
Child was fed food or fluids other than breast milk in first 6 months^+^	Yes	9371	347	0.71 (0.60–0.84)	0.000
	Unknown	197	9		

^∗^Parental response to the question: “Has your child ever had a strong allergic reaction to peanuts/peanut products, and/or other nuts/nut products?”

^#^Adjusted for age and sex where appropriate.

^+^Includes children who were breast fed and given other food/fluid, and those who were exclusively given other food/fluid.

## References

[1] Grundy J, Matthews S, Bateman B, Dean T, Arshad SH (2002). Rising prevalence of allergy to peanut in children: data from 2 sequential cohorts. *Journal of Allergy and Clinical Immunology*.

[2] Hughes DA, Mills C (2001). Food allergy: a problem on the increase. *Biologist*.

[3] Mullins RJ, Dear KBG, Tang MLK (2009). Characteristics of childhood peanut allergy in the Australian Capital Territory, 1995 to 2007. *Journal of Allergy and Clinical Immunology*.

[4] World Health Organisation (WHO) Infant feeding recommendation (as stated in the Global Strategy on Infant and Young Child Feeding).

[5] Des Roches A, Paradis L, Singer S, Seidman E (2005). An allergic reaction to peanut in an exclusively breastfed infant. *Allergy*.

[6] Gerrard JW, Perelmutter L (1986). IgE-mediated allergy to peanut, cow’s milk, and egg in children with special reference to maternal diet. *Annals of Allergy*.

[7] Frank L, Marian A, Visser M, Weinberg E, Potter PC (1999). Exposure to peanuts in utero and in infancy and the development of sensitization to peanut allergens in young children. *Pediatric Allergy and Immunology*.

[8] Koplin JJ, Osborne NJ, Wake M (2010). Can early introduction of egg prevent egg allergy in infants? A population-based study. *Journal of Allergy and Clinical Immunology*.

[9] Miŝak Z (2011). Infant nutrition and allergy. *Proceedings of the Nutrition Society*.

[10] Cochrane S, Beyer K, Clausen M (2009). Factors influencing the incidence and prevalence of food allergy. *Allergy*.

[11] Nwaru BI, Erkkola M, Ahonen S (2010). Age at the introduction of solid foods during the first year and allergic sensitization at age 5 years. *Pediatrics*.

[12] Greer FR, Sicherer SH, Burks AW (2008). Effects of early nutritional interventions on the development of atopic disease in infants and children: the role of maternal dietary restriction, breastfeeding, timing of introduction of complementary foods, and hydrolyzed formulas. *Pediatrics*.

[13] Anderson J, Malley K, Snell R (2009). Is 6 months still the best for exclusive breastfeeding and introduction of solids? A literature review with consideration to the risk of the development of allergies. *Breastfeeding Review*.

[14] Prescott SL, Smith P, Tang M (2008). The importance of early complementary feeding in the development of oral tolerance: concerns and controversies. *Pediatric Allergy and Immunology*.

[15] Zutavern A, Brockow I, Schaaf B (2008). Timing of solid food introduction in relation to eczema, asthma, allergic rhinitis, and food and inhalant sensitization at the age of 6 years: results from the prospective birth cohort study LISA. *Pediatrics*.

[16] Kemp AS (2005). Severe peanut allergy in Australian children. *Medical Journal of Australia*.

[17] Phillips CB, Yates R, Glasgow NJ, Ciszek K, Attewell R (2005). Improving response rates to primary and supplementary questionnaires by changing response and instruction burden: cluster randomised trial. *Australian and New Zealand Journal of Public Health*.

[18] Hourihane JO, Aiken R, Briggs R (2007). The impact of government advice to pregnant mothers regarding peanut avoidance on the prevalence of peanut allergy in United Kingdom children at school entry. *Journal of Allergy and Clinical Immunology*.

[19] Prescott SL, Tang MLK (2005). The Australasian Society of Clinical Immunology and Allergy position statement: summary of allergy prevention in children. *Medical Journal of Australia*.

[20] Vadas P, Wai Y, Burks W, Perelman B (2001). Detection of peanut allergens in breast milk of lactating women. *Journal of the American Medical Association*.

[21] Sicherer SH, Wood RA, Stablein D (2010). Maternal consumption of peanut during pregnancy is associated with peanut sensitization in atopic infants. *Journal of Allergy and Clinical Immunology*.

[22] Desroches A, Infante-Rivard C, Paradis L, Paradis J, Haddad E (2010). Peanut allergy: is maternal transmission of antigens during pregnancy and breastfeeding a risk factor. *Journal of Investigational Allergology and Clinical Immunology*.

[23] Uenishi T, Sugiura H, Tanaka T, Uehara M (2011). Aggravation of atopic dermatitis in breast-fed infants by tree nut-related foods and fermented foods in breast milk. *Journal of Dermatology*.

[24] Sicherer SH, Burks AW, Sampson HA (1998). Clinical features of acute allergic reactions to peanut and tree nuts in children. *Pediatrics*.

[25] Kljakovic M, Gatenby P, Hawkins C (2009). The parent-reported prevalence and management of peanut and nut allergy in school children in the Australian Capital Territory. *Journal of Paediatrics and Child Health*.

[26] Hourihane JOB, Dean TP, Warner JO (1996). Peanut allergy in relation to heredity, maternal diet, and other atopic diseases: results of a questionnaire survey, skin prick testing, and food challenges. *British Medical Journal*.

[27] Lack G, Fox D, Northstone K, Golding J (2003). Factors associated with the development of peanut allergy in childhood. *New England Journal of Medicine*.

[28] Peeters KABM, Knulst AC, Rynja FJ, Bruijnzeel-Koomen CAFM, Koppelman SJ (2004). Peanut allergy: sensitization by peanut oil-containing local therapeutics seems unlikely. *Journal of Allergy and Clinical Immunology*.

[29] Joseph CLM, Ownby DR, Havstad SL (2011). Early complementary feeding and risk of food sensitization in a birth cohort. *Journal of Allergy and Clinical Immunology*.

[30] Sariachvili M, Droste J, Dom S (2010). Early exposure to solid foods and the development of eczema in children up to 4 years of age. *Pediatric Allergy and Immunology*.

